# Fighting the waves; Covid-19 family life interference in a neurodevelopmental disorder-caregiver population

**DOI:** 10.1186/s12913-022-07836-3

**Published:** 2022-04-10

**Authors:** Mats Nylén-Eriksen, Mariela Loreto Lara-Cabrera, Ellen Karine Grov, Hanne Skarsvaag, Irene Lie, Tone Dahl-Michelsen, Torill Margaret Sæterstrand, Arthur Mandahl, Hege Hafstad, Mona Breding Lersveen, Ann Kristin Bjørnnes

**Affiliations:** 1 Institute of Nursing and Health Promotion, Oslo Metropolitan University, Oslo, Norway; 2 Department of Research and Development, Division of Mental Health, St Olav’s University Hospital, Trondheim, Norway; 3 Faculty of Medicine and Health Sciences, Department of Mental Health, Norwegian University of Science and Technology (NTNU), Trondheim, Norway; 4 Division of Psychiatry, Tiller Community Mental Health Centre, St. Olav’s University Hospital, Trondheim, Norway; 5 ADHD Norway, Oslo, Norway; 6 Department of Cardiothoracic Surgery, Division of Cardiovascular and Pulmonary Diseases, Center for Patient-Centered Heart and Lung Research, Oslo UniversityHospital, Oslo, Norway; 7 Institute of Physiotherapy, Oslo Metropolitan University, Oslo, Norway; 8 Vårres Regional User-Led Center Central-Norway, Trondheim, Norway

**Keywords:** COVID-19, Neurodevelopmental disorder, NDD, ADHD, ASD, Informal caregiver, Family life interference, Family life

## Abstract

**Introduction:**

The current COVID-19 pandemic interferes with family lives across the world, particularly families of children with neurodevelopmental disorders (NDDs) are at a greater risk for being negatively impacted by the pandemic. Together with representatives from this caregiver population the aim was to explore the interference associated with normal family life caused by the COVID-19 pandemic.

**Method:**

This is a descriptive study using a cross-sectional design. Following a strategic network sampling strategy, a user-developed national survey was completed by a larger sample (*N* = 1,186) of parents and informal caregivers of children with NDDs. The survey utilized a combination of both closed and open-ended questions, and a logistic regression analysis was carried out to assess the association between family characteristics, characteristics of the child, and COVID-19 related family life interference. Before carrying out the regression an inductive content analysis of the open-ended question on `How has the isolation affected the family´ was carried out to construct the outcome variable.

**Results:**

The initial analysis indicated that the COVID-19 pandemic induced a shift in everyday family life and a lack of guidance and support related to managing the challenges they were facing. Caregivers who reported that COVID-19 had significantly interfered with their family life, were more likely to report having anxious children, and to have experienced an increased number of conflicts at home. The logistic regression showed that both anxious children and increased conflicts considerably increased the risk for reporting family life interference compared to those that reported no increased conflicts or anxious children.

**Discussion:**

Considering how the COVID-19 related increased conflicts at home and anxious children threaten the family life of the NDD caregiver population, as an external source of family stress, which might lead to negative impact on their mental and physical well-being, the need for further research in collaboration with user representatives is apparent. Our study suggests that more information should be provided to healthcare providers, social professionals, peers, people with NDDs, and caregivers of people with NDDs about the potential threats that a stressful life event such as the current pandemic can pose to their mental and physical health and their family life.

**Supplementary Information:**

The online version contains supplementary material available at 10.1186/s12913-022-07836-3.

## Introduction

The current COVID-19 pandemic has interfered with the family lives of informal caregivers of children across the world, threatening their well-being [1]. A recent report (*N* = 2,500) on informal caregivers in 16 countries revealed that 90.6% of the respondents were concerned about how their caring role and their personal and caring circumstances were affected by the COVID-19 pandemic. The report shows how the COVID-19 pandemic is interfering with the carer´s life and negatively impacting their quality of life (78.9%) and mental health (66.5%) [2]. However, for some families, the interference caused by the COVID -19 pandemic is more extensive and may have different consequences for them than it does for the majority of the population [3]. Measures to reduce contact between individuals, such as the closure of educational and childcare establishments affect the daily routines and roles of families and may result in considerable psychosocial costs [4]. Psychosocial impacts related to the reduction of contact between individuals [5] have extended beyond disease resolution in past outbreaks [6]. Findings from a recent scoping review [7] including 29 articles show that isolation and home quarantine interfere with the family life of families with children with neurodevelopmental disorders (NDDs) due to routine disruption resulting in increased parental stress and threaten their wellbeing. Interference occurs where there is a role overload and role conflict, a situation in which an individual’s total role obligations are overly demanding, and the adequate performance of one role endanger the adequate performance of other roles [8], and newer studies indicate that families and parents are disproportionately vulnerable [5, 7].

Families of children with NDDs such as autism spectrum disorder (ASD) or attention-deficit/hyperactivity disorder (ADHD) and Tourette’s syndrome are at particular risk during the COVID-19 pandemic [9]. In Norway, the estimated percentage of children suffering from one or more NDDs is > 5% [10], assuming each private household have one child with NDD, at a minimum it could represent as many as 37,762 private households [11]. A recent Norwegian report on caregivers (National caregiver survey) [12] finds that families located in urban areas, which were subjected to the harshest restrictions related to the pandemic, experienced a higher level of COVID-19 interference, and a heavier caregiver burden than families without children with health challenges. The caregivers who live in more rural areas reported to a greater extent than caregivers in the more urban areas that they lack health service offers particularly for them. The report also showed that even though they are caregivers of children who to the greatest extent utilize service offerings and support schemes, this caregiver group experience low availability of such services [12]. NDDs are a group of disorders affecting the development and function of the brain [13]. Individuals with NDDs are particularly vulnerable to the distress induced by the COVID-19 pandemic infection control and prevention measures, and to stress caused by the unpredictability of the situation [14, 15]. As a result, they may display increased behavioural, mental, and physical problems [14, 16]. The immediate stressful impact and the unpredictability associated with the first period of the pandemic may have caused difficulty with executive function skills in individuals with ADHD [17] and ASD [14], due to the challenges they face with cognitive flexibility and inhibitory control. Executive functions are an umbrella term for mental processes which are important in every aspect of an individual’s life, and consist of three core functions: inhibitory control, work memory, and cognitive flexibility. Dysfunction in one or more of these functions will lead to difficulties in taking care of one's health [18]. NDDs such as ADHD and ASD are characterized by executive dysfunction [19], and are associated with reduced mental and physical health, quality of life, school/job performances, and marital or -family functioning [18].

ASD and ADHD [20] are associated with co-morbidities such as behavioural problems, anxiety, or depression. In addition, children diagnosed with ADHD and ASD are particularly sensitive to changes in routine and restrictions to activity and are therefore particularly vulnerable during the COVID-19 pandemic because of the associated lockdowns of kindergarten and school. Parents of children with NDDs often report an above-average rate of mental health issues [21–23] and the involvement of hereditary factors in certain NDDs (e.g., ADHD) may suggest that some caregivers have the diagnosis themselves [24]. This implies that these individuals may experience increased stress and caregiver burden. Parenting a child with an NDD was associated with an increased level of caregiver burden even before the global COVID-19 pandemic [23, 25, 26]. Results from a European study (*N* = 2,326 caregivers) demonstrated strains related to work, social activity, family life, and increased parental worry and stress among caregivers of children and adolescents with ADHD [27]. Similar findings were reported for caregivers of children and adolescents with ASD in a separate study [22]. Conflict levels are higher in families of children with ADHD [21] and ASD [22] when compared with control families, and parents in these families often report an unmet need for respite care, caregiving support, and accessible childcare [25]. Considering the findings of previous research on these stressors on informal caregivers [1], it is reasonable to expect an even higher level of burden during the current COVID-19 pandemic as a result of increased caregiver responsibilities.

It is reasonable to assume that during lockdowns of school, kindergarten, and other activities, children with NDD are subject to an increased risk that their needs related to school and psychosocial functioning—which would normally be handled by the kindergarten, school, or another public service such as respite care—will not be met [28]. As a result, the informal caregivers of these children were presumably forced into new and different roles to meet their child’s needs, while also experiencing role changes in their professional work life (e.g., working from home, temporarily being laid off, or losing their job) [3]. It is conceivable that this may lead to a role overload, which could lead to conflict (i.e., COVID-19 family life interference). We understand the COVID-19 family life interference as a result of the routine disruption and increased external stress on the caregivers and their children caused by the isolation and home quarantine due to the COVID-19 pandemic [29], thus we hypothesize that the COVID-19 pandemic will interfere with the family life of NDD caregivers and their children, as an external source of family stress.

To our knowledge, there is currently no research available on family life interference caused by the COVID-19 pandemic among the NDD caregiver population and there is a need for more research on the subject of family life in families with children with NDD during the COVID-19 pandemic [7, 30]. Together with representatives from this caregiver population we aim to explore the interference associated with normal family life caused by the COVID-19 pandemic, in an NDD caregiver population during the first lockdown in Norway. Our specific objectives include: 1) to describe COVID-19 related interference with normal family life and the impact on children with NDDs, and 2) to explore the associations between family characteristics, characteristics of the child, and COVID-19 related family life interference.

## Materials and methods

### Study design

This is a descriptive study using a cross-sectional design. Based on an extensive review of the literature we developed several hypotheses demonstrating our specific expectation to our findings, which is presented in a summarizing table along with the hypothesis testing and decision in the result section (Table 4).

### Sample and procedure

This cross-sectional study uses data from a user-developed national survey among parents and informal caregivers of children with NDDs. The web-based survey was conducted from late April to mid-May 2020 by Vårres brukerstyrt senter Midt Norge (Vårres Regional User-led Center Central-Norway [Vårres]). Vårres’ core tasks are to collect and disseminate user- and informal caregiver experiences from their target groups within the fields of mental health and substance abuse. The cooperation with different organizations within these fields provides unique access to participants of the survey. Following a strategic network sampling with the “seeds” being the cooperating organizations represented the diagnostic groups; ADHD, Autism Spectrum Disorders, and Tourette Syndrome, commonly referred to as NDDs, disseminating the written information about the survey and the participating link to the survey to their members through their social media accounts and web pages. The same information was distributed on Vårres webpage, as well as on social media accounts. The recruitment of the respondents avoided first-hand contact with the researcher. The information and link shared on social media and webpages aimed to recruit informal caregivers of at least one child with NDDs from all of Norway´s 11 counties. Consent was given by clicking to indicate acceptance to participate in the survey. Then, after completing the survey questions, participants clicked again to submit their answers. This is further explained under the section ‘Ethics statement and consent to participate’. The survey was conducted using the survey software `Easy Quest` [31] which facilitates to a secure and anonymous survey. With guidance from a researcher knowledgeable about NDDs, the questions were prepared by employees in Vårres with lived experience as informal caregivers for children with NDDs, as well as extensive course and information work involvement with the target group through various public health agencies. The employees (i.e., the users/user-organization) had the final say in the inclusion/exclusion of survey questions. The survey was open between April 27 and May 20.

### Measures

Information about the characteristics of the families and the children were collected using a demographic form, which included questions about location, income, and if they had additional children younger than 18 years living at home. The number of questions related to the demographic characteristics was purposefully kept low to ensure anonymity. The rest of the survey used a combination of both closed and open-ended questions, focusing on three main areas: 1) parents work situations, 2) child’s school situation (e.g., home-schooling or not), and support network currently and prior to the COVID-19 pandemic, and 3) if the social distancing had negatively affected the child and the family (e.g., increased child anxiety and conflict in the family). The user representatives recommended to limit the response options to accommodate potential challenges with executive functions among the respondents [24]. The respondents were asked to indicate their answers to the closed-ended questions by choosing between two (i.e., ‘yes’ or ‘no’ response) or three ordinal levels (i.e., answered ‘yes’, ‘to some extent’, ‘no’).

Using open-ended questions respondents were also asked if there was something positive associated with the current situation. The combination of both closed and open-ended questions was purposefully used to achieve the study´s objectives. The open-ended questions were used to counteract the sparsely available knowledge regarding the range of possible answers on several of the questions due to the unprecedented challenges caused by the COVID-19 pandemic and lack of existing validated measures.

### Data analysis

Data were analysed using IBM SPSS (Statistical Package for the Social Sciences) Statistics 27 for Windows. Pearson Chi-Square (χ^2^) was used to assess significance for the categorical variables, which included all close-ended survey questions (i.e., questions only answered ‘yes’, ‘to some extent’, ‘no’). A logistic regression analysis with a backward elimination procedure [32] was carried out to assess the association between family characteristics, characteristics of the child, and COVID-19 related family life interference. Since the caregiver support provided may differ between rural and urban areas, it was conceivable that caregivers in the included counties might have experienced different degrees of COVID-19 family life interference. County was included as a correlate of the outcome in the logistic regression. Before carrying out the backward logistic regression an inductive content analysis was carried out to construct the outcome variable.

### Inductive content analysis

To gain new insight and build up categories describing the phenomenon, the open-ended survey question related to COVID-19 family life interference were systemized using inductive content analysis [33]. Two of the authors (MNE, AKB) carried out the analysis. The initial question was “How has the isolation affected the family”, the two authors then systematically analysed all the answers to the open-ended question ending up with two clear categories derived from the data. The data was then coded into the two groups ‘Family Life Interference’ and ‘No Family Life Interference’. The COVID-19 Family Life Interference’ variable was cross-checked against other variables to ensure correct coding. Table 1 presents quotes that emerged from the open-ended question representing the two categories derived from the data.

**Table 1:** Construction of the COVID-19 family life interference output variable

Quantitative categories	Example quote:
**Family life interference**	“Higher levels of conflict, everyone is tired. We as parents have to juggle work, studies, and follow-up of our children are severely affected psychologically.” “My partner avoids coming home from work, so I am left with the whole job alone. I think the child and I have managed something good. Some days are heavier than others, but it helps when the sun peeks out.” “Very bad. Everything is at the breaking point!!!” “For the better for the child with problem. Soon, the rest of the family will no longer survive.” “Many conflicts between everyone. Meltdowns, tantrums, smashing of houses and objects.” “We are alone. Our child is lonely.” “Parents have lost their much needed “breathing room” as a result of being together 24/7.” “Increased conflict level, more strain on caregivers who must carry out new roles such as being a teacher, in addition to doing our normal jobs in home office.” “Hopeless chaos within the house´ four walls.” “Catastrophically. Nearing marital breakdown and sibling conflict increased to an unmanageable level.”
**No family life interference**	“We've had more time together.” “The family has become closer.” “We have a calmer and more harmonious everyday life.” “Exclusively positive.” “We have played more. Collaborate better. Talked more and relaxed more. Nice to get more insight into the schoolwork.” “It has gone well. We are a family that is used to being together and at home from before.” “This has been good to us, experienced less stress due to more flexible time management.” “Nothing has changed.” “For the better. Child has been doing great work in home school.” “Pretty good actually.” “Increased familial unity, many good family activities and more time together.”

We systemized the answers to the open-ended question; “How has the isolation affected you as a family” into the two categories “family life interference” and “no family life interference” using inductive content analysis

### Backward logistic regression

None of the independent variables had a missing value of > 2%, and the dependent variable had a missing value of 19%. We assumed that the missing data were missing at random (MAR) and used casewise deletion in the regression analysis, which is the most common approach to dealing with missing data in observational studies [34]. Variables were manually dropped step-by-step according to both significance level and a careful evaluation of clinical plausibility in accordance with recommendations from Hosmer, Lemeshow, and Studivant [32]. The model's explanation of family life interference was examined by Cox-Snell R-squared and Nagelkerke R-squared. The Hosmer–Lemeshow goodness of fit test was used to examine how well the final model matched the data.

## Results

The study sample (*N* = 1,186) is comprised of caregivers of children with NDD from all 11 counties in Norway (Table 2). Regarding socioeconomic consequences, 53% (*n* = 628) of the caregivers worked from a home office, 85% (*n* = 1,002) reported that they did not lose any income, and 75% (*n* = 892) reported no concerns about the economy during the Covid-19 lockdown in spring 2020. Fifty-seven percent (*n* = 673) of the caregivers had to stay at home in order to help their child while the school was closed, and 70% (*n* = 831) reported having more than one child in school or kindergarten at home, which had to stay at home. The majority (65%, *n* = 774) recounted that the school provided them with equipment and aids to enable the child to follow the home-schooling program. Contrastingly, the majority of the caregivers (80%, *n* = 945) did not receive any guidance (e.g., oral or written) during this lockdown to help their child with the home-schooling, and 83% (*n* = 986) reported that they did not get any support or guidance from the school, the Child and Adolescents Mental Health Services (CAMHS) or other public agencies to handle the everyday life during the lockdown. Forty-five percent (*n* = 533) of the children reported anxiety associated with the closure of the school in this period, and only 31% (*n* = 366) of the children had a respite care scheme (e.g., respite care in the home or at an institution). Increased conflicts at home due to the closure of the school were reported for 56% (*n* = 665) of the caregivers. Fifty-one percent (*n* = 488) of the caregivers reported that the Covid-19 lockdown and isolation affected their family life in a negative way.

**Table 2:** The distribution of the independent variables’ frequencies from the total sample (*N* = 1186) and on the dependent variable “Family life interference” (*n* = 956)^a^﻿

	**Total sample (*N* = 1186)**	**No family life interference (*n* = 468)**	**Family life interference (*n* = 488)**	**P-value**
	**n (%)**	**n (%)**	**n (%)**	
**County**				0.261
Oslo Viken Møre and Romsdal Agder Rogaland Trøndelag Vestland Nordland Troms and Finnmark Vestfold and Telemark Innland Not responded	100 (8.4) 222 (18.7) 72 (6.1) 57 (4.8) 103 (8.7) 91 (7.7) 132 (11.1) 40 (3.4) 52 (4.4) 76 (6.4) 79 (6.7) 162 (13.7)	32 (6.8) 91 (19.4) 29 (6.2) 20 (4.3) 47 (10) 38 (8.1) 54 (11.5) 18 (3.8) 20 (4.3) 36 (7.7) 35 (7.5) 48 (10.3)	55 (11.3) 88 (18) 33 (6.8) 25 (5.1) 34 (7) 36 (7.4) 48 (9.8) 21 (4.3) 22 (4.5) 26 (5.3) 35 (7.2) 65 (13.3)	
**Home office during shutdown**				0.760
No Yes	550 (46.4) 628 (53)	204 (43.9) 261 (56.1)	218 (44.9) 268 (55.1)	
**Lost income during shutdown**				0.006
No Yes	1002 (84.5) 180 (15.2)	418 (89.3) 50 (10.7)	405 (83.2) 82 (16.8)	
**Concerned about economy**				0.000
No Yes	892 (75.2) 290 (24.5)	390 (83.3) 78 (16.7)	347 (71.3) 140 (28.7)	
**Must stay home to help child while** **school is closed**				0.000
No Yes	508 (42.8) 673 (56.7)	221 (47.4) 245 (52.6)	166 (34.2) 320 (65.8)	
**School has arranged with equipment and aids for child to be able to follow home schooling**				0.097
No Yes	401 (33.8) 774 (65.3)	150 (32.1) 317 (67.9)	181 (37.2) 305 (62.8)	
**School facilitates for educational support for child during home schooling**				0.008
No Partially Yes	401 (33.8) 514 (43.3) 260 (21.9)	143 (30.6) 202 (43.3) 122 (26.1)	177 (36.3) 222 (45.6) 88 (18.1)	
**Parent have received guidance along the way to help the child during home schooling**				0.022
No Yes	945 (79.7) 218 (18.4)	359 (77.5) 104 (22.5)	403 (83.4) 80 (16.6)	
**Parent have received support or guidance from the school, CAPU**^**b**^** or others to handle everyday life during the shutdown**				0.202
No Yes	986 (83.1) 186 (15.7)	396 (85.2) 69 (14.8)	399 (82.1) 87 (17.9)	
**The closure of the school has made the child anxious**				0.000
No A little Yes	641 (54) 355 (29.9) 178 (15)	321 (68.6) 117 (25) 30 (6.4)	202 (41.6) 163 (33.5) 121 (24.9)	
**More than one child in school or kindergarten at home**				0.596
No Yes	346 (29.2) 831 (70.1)	136 (29.1) 331 (70.9)	134 (27.6) 352 (72.4)	
**The closure of the school and kindergarten contributed to increased conflicts at home**				0.000
No A little Yes	512 (43.2) 322 (27.2) 343 (28.9)	323 (69) 104 (22.2) 41 (8.8)	88 (18.1) 156 (32.1) 242 (49.8)	
**Child has a respite care scheme**				0.000
No Yes	810 (68.3) 366 (30.9)	346 (74.2) 120 (25.8)	293 (60) 195 (40)	

^a^The 230 respondents missing from the output variable is due to lack of answering the open-ended question. ^b^Child and Adolescent Psychiatry Unit [in Norwegian BUP]. The p-values were derived from the Pearson Chi-square test for independence

### COVID-19 family life interference

A Chi-square test (χ^2^) for independence indicated several areas where caregivers experiencing family life interference differed significantly from those reporting no family life interference. They reported a larger decline in income (*p* = 0.006), they were more concerned about their economy (*p* = 0.000), were more often required to stay at home to help their child (*p* = 0.000), experienced less facilitation from the school regarding educational support for their child(ren) during home-schooling (*p* = 0.008), and received less support from CAMHS or other public agencies (*p* = 0.022), compared to caregivers reporting no family interference. Concerningly, these caregivers reported a higher number of children having a respite care scheme (*p* = 0.000), their children were more anxious (*p* = 0.000), and the closure of the school and kindergarten contributed to an increased number of conflicts at home (*p* = 0.000) compared to those reporting a more positive family outcome under the lockdown.

The logistic regression analysis indicated that increased conflict at home was associated with approximately nineteen times greater risk for reporting family life interference compared with those experiencing no conflicts (OR = 19.04, 95% CI = 12.34–29.37, *p* < 0.001). Concerningly, even a minor increase in conflict at home was associated with almost a five times increased risk (OR = 4.91, 95% CI = 3.42–6.72, *p* < 0.001). The caregivers with children who became anxious as a result of the school closure had two times increased risk of experiencing family interference (OR = 2.79, 95% CI = 1.67–4.677, *p* < 0.001) compared to the caregivers of children without anxiety. Families with children having a respite care scheme had 57% increased risk for a negative outcome (OR = 1.57, 95% CI = 1.12–2.21, *p* = 0.009), and concerns about economy were associated with an 83% increased risk (OR = 1.83, 95% CI = 1.24–2.70, *p* = 0.002) Residing in Vestfold and Telemark (OR = 0.71, 95% CI = 0.13-0.68, *p* = 0.004), as well as in Rogaland (OR = 0.44, 95% CI = 0.20-0.94, *p* = 0.033) and was associated with decreased risk for family life interference compared to the caregivers residing in Oslo (Tables 3 and 4).

**Table 3:** Logistic Regression; Backward Stepwise elimination

	**Unadjusted model**	**Step 1**	**Step 2**	**Step 3**	**Step 4**	**Step 5**
	**OR [95%CI]**	**OR [95%CI]**	**OR [95%CI]**	**OR [95%CI]**	**OR [95%CI]**	**OR [95%CI]**
**County (ref.)**						
Viken	.563* [.333, .951]	.577 [.299, 1.113]	.549 [.286, 1.053]	.585 [.307, 1.155]	.579 [.305, 1.098]	.578 [.305, 1.096]
Møre and Romsdal	.662 [.341, 1.284]	.863 [.380, 1.960]	.838 [.370, 1.895]	.840 [.4374, 1.890]	.788 [.354, 1.753]	.783 [.353, 1.740]
Agder	.727 [.350, 1.512]	.767 [.305, 1.929]	.718 [.286, 1.802]	.752 [.303, 1.868]	.763 [.307, 1.897]	.755 [.304, 1.879]
Rogaland	.421* [.226, .783]	.475 [.218, 1.036]	.447* [.207, .969]	.455* [.211, 980]	.439* [.204, .944]	.436* [.203, .937]
Trøndelag	.551* [.293, 1.036]	.560 [.253, 1.243]	.519 [235, 1.144]	.546 [.250,1.191]	.530 [.244, 1.151]	.528 [.243, 1.148]
Vestland	.517* [.288, .927]	.595 [.288, 1.231]	.563 [274, 1.159]	.575 [.281, 1.179]	.567 [.277, 1.161]	.568 [.278, 1.161]
Nordland	.679 [.316, 1.460]	.729 [.272, 1.953]	.692 [.259, 1.847]	.774 [.295, 2.033]	.835 [.326, 2.138]	.831 [.324, 2.134]
Troms and Finnmark	.640 [.304, 1.350]	.724 [.289, 1.813]	.715 [.287, 1.781]	.676 [.277, 1.652]	.668 [.273, 1.633]	.679 [.278, 1.654]
Vestfold and Telemark	.420* [.216, .818]	.302* [.129, .705]	.291** [.126, .675]	.311** [.135, .715]	.295** [.129, .676]	.297** [.130, .680]
Innlandet	.582 [.307, 1.103]	.789 [.346, 1.795]	.748 [.332, 1.687]	.750 [.340, 1.654]	.759 [.344, 1.671]	.714 [.326, 1.561]
Not responded	.788 [.444, 1.398]	1.066 [.529, 2.150]	1.017 [.507, 2.040]	.1.029 [.515, 2.056]	1.049 [.526, 2.089]	1.041 [.523, 2.070]
**Concerned about economy**	2.017*** [1.476, 2.757]	1.761* [1.141, 2.717]	1.853** [1.244, 2.761]	1.797** [1.215, 2.658]	1.819** [1.231, 2.688]	1.828** [1.237, 2.701]
**Anxious child (ref.)**						
A little	2.214*** [1.647, 2.975]	1.526 *[1.062, 2.194]	1.548* [1.079, 2.222]	1.537* [1.074, 2.199]	1.591* [1.116, 2.269]	1.606* [1.127, 2.289]
Yes	6.409*** [4.141, 9.920]	2.657*** [1.572, 4.491]	2.601*** [1.547, 4.373]	2.688*** [1.604, 4.504]	2.758*** [1.647, 4.618]	2.796*** [1.671, 4.677]
**Increased conflict (ref.)**						
A little	5.506*** [3.910, 7.752]	4.915*** [3.376, 7.156]	4.880*** [3.362, 7.085]	4.848*** [3.357, 7.000]	5.038*** [.3.500, 7.252]	4.914*** [3.422, 6.716]
Yes	21.665*** [14.430, 32.525]	17.985*** [11.507, 28.110]	17.805*** [11.431, 27.734]	18.077*** [11.629, 28.100]	19.555*** [12.617, 30.307]	19.041*** [12.344, 29.372]
**Child has a respite care scheme**	1.919*** [1.457, 2.528]	1.574* [1.107, 2.239]	1.548* [1.089, 2.199]	1.585* [1.121, 2.241]	1.545* [1.097, 2.176]	1.572* [1.117, 2.213]
**More than one child in school or kindergarten**	1.079 [.814, 1.431]	.755[.524, 1.088]	.756 [.526, 1.086]	.786 [.549, 1.125]	.831 [.584, 1.184]	
**Stay home—Home schooling**	1.739*** [1.339, 2.258]	1.317 [.926, 1.874]	1.352 [.954, 1.918]	1.233 [.887, 1.715]		
**No parental guidance home school**	1.459* [1.055, 2.018]	1.416 [.890, 2.254]	1.482 [.958, 2.294]	1.259 [.842, 1.882]		
**Home office**	.961 [.744, 1.241]	.841 [.592, 1.195]	.831 [.587, 1.178]			
**No parental support or guidance to handle everyday life**	.799 [.566, 1.129]	.709 [.434, 1.159]	.724 [.446, 1.174]			
**School did not arrange with equipment**	1.254 [.960, 1.639]	.837 [.581, 1.207]				
**Educational support for child (ref.)**						
Partly	.888 [.663, 1.188]	1.029 [.697, 1.518]				
Yes	.583** [.410, .828]	.764 [.480, 1.316]				
**Lost income**	1.693** [1.161, 2.468]	1.194 [.707, 2.016]				

^*^p < 0.05; **p < 0.005; ***p < 0.001; Hosmer and Lemeshow test; *p* = step1:0.910 step2:0.870 step3:0.779 step4:0.900 step5:0.730 Coding of categorical variables; County (ref.) = Oslo, Educational support for child (ref.) = No, Anxious child (ref.) = No, Increased conflict (ref.) = No, Concerned about economy; 1 = Yes 0 = No, Child has a respite care scheme; 1 = Yes 0 = No, More than one child in school or kindergarten; 1 = Yes 0 = No, stay home – home schooling; 1 = Yes 0 = No, No parental guidance home schooling; 1 = No 0 = Yes, home office; 1 = Yes 0 = No, No parental support or guidance to handle everyday life; 1 = No 0 = Yes, school did not arrange with equipment; 1 = No 0 = Yes, lost income; 1 = Yes 0 = No

**Table 4:** Hypothesis testing and decision

	Null Hypothesis	Test	Sig	Decision
1	There is no difference between the families experienced COVID-19 family life interference and those that did not regarding their financial concerns due to lost income as a result of the lockdown	Pearson Chi-square (χ^2^)	0.000	Reject the null hypothesis
2	There is no difference between the families experienced COVID-19 family life interference and those that did not regarding either increased conflict at home or anxious child as a result of the lockdown (i.e., closing of school, kindergartens)	Pearson Chi-square (χ^2^)	0.000	Reject the null hypothesis
3	There is a difference between the families experienced COVID-19 family life interference and those that did not regarding either ‘the lockdown resulting in staying home to home-school their child’, ‘the schools/kindergarten facilitating for educational support for the child’ or ‘the caregivers receiving support and guidance on how to help their child’ during the home-schooling	Pearson Chi-square (χ^2^)	0.000 0.008 0.022	Reject the null hypothesis
4	There is no association between which county the respondents reside in and COVID-19 family life interference	Backwards logistic regression	*p* < 0.05	Reject the null hypothesis
5	There is no association between financial concerns and COVID-19 family life interference	Backwards logistic regression	*p* < 0.05	Reject the null hypothesis
6	There is no association between either increased conflict at home or anxious child as a result of the lockdown (i.e., closing of school, kindergartens) and COVID-19 family life interference	Backwards logistic regression	*p* < 0.05	Reject the null hypothesis
7	There is no association between either ‘the lockdown resulting in staying home to home-school their child’, ‘the schools/kindergarten facilitating for educational support for the child’ or ‘the caregivers receiving support and guidance on how to help their child’ during the home-schooling and COVID-19 family life interference	Backwards logistic regression	*p* > 0.05 *p* > 0.05 *p* > 0.05	Retain the null hypothesis

## Discussion

The purpose of the present study was to explore the interference associated with family life and the COVID-19 pandemic in an NDD caregiver population during the first social lockdown in Norway.

The results indicate that the COVID-19 pandemic induced a shift in everyday family life, which was characterized by abruptions, changes, disruptions, and ongoing insecurity for approximately one-third of the sample. Most of the sample did not receive any guidance on how to help their children with home-schooling, nor did they receive any guidance or support on how to handle everyday life during the lockdown. This is a concern since distorted caring positions exhaust and drain the family relationship [3, 35] Considering the challenges regarding executive dysfunction in children with NDDs [14, 17], higher rates of mental health issues [36] and possible role overload [3] among their caregivers, our findings regarding high rates of reported anxiety in the children, and increased conflict at home might reflect external family stress caused interference with the family life resulting from the COVID-19 pandemic. Recently published studies reported similar findings regarding the psychological impact of COVID-19 on family life in an NDD caregiver population as a result of the lockdown, isolation, and changes in daily routines [3, 5, 7, 37].

The results from the logistic regression analysis presented several concerning results indicating the vulnerability of the NDD-caregiver population. Both anxiety- and conflict level during the lockdown were important concerns among our user representatives and was included in the questionnaire to capture key facets of the caregiver experience. COVID-19 related family life interference was associated with approximately nineteen times greater risk for increased conflict at home, and even a minor increase in conflict at home was associated with five times increased risk compared with those reporting no conflict. Considering that the COVID-19 pandemic increases familial conflicts such as sibling conflicts, especially in families with children suffering from special educational needs and disabilities and how these increased conflicts increase parental distress [37] our findings are even more concerning. Almost half the children reacted with anxiety to the changes induced by the social lockdown, which increased the risk of family interference by two times compared with caregivers of children without anxiety. Considering the extent to which the closure of educational and childcare establishments as a result of the COVID-19 pandemic interferes with the daily lives, tasks, and routines of children and caregivers with NDD and their families [14, 15, 37], these findings may illustrate the ramification of living under the strict restrictions associated with the pandemic for vulnerable groups such as the NDD caregiver population. Our findings suggest that both children and caregivers, experienced challenges in adapting to a new, isolated, digital, and unpredictable everyday life in which their roles and expectations have changed. This may be due to executive dysfunctions in cognitive flexibility and inhibitory control (i.e., appropriately adjusting behaviour and cognition to adequately meet new demands, roles, or priorities within a changing environment, such as the current COVID-19 pandemic) [18, 19]. In addition, one must keep in mind that hereditary factors reflect research on NDDs [24], thus, it is conceivable that some caregivers participating in the study might have a NDD themselves. This potentially complicates the situation further and increases their need for support and guidance. The findings suggest that COVID-19 interferes with family life by causing uncertainty about what the future brings, forcing a transition to digital platforms at work and at school, and exposing people to divergent information and that this has led to increased unrest and greater difficulties with executive functions among people with NDDs. For caregivers, the results suggest an increased threat to their mental health, as a result of increased caregiver burden and familial stress due to COVID-19 family life interference, along with a threat to their resilience in dealing with the consequences of the COVID-19 pandemic and the negative impact it has had on their mental and physical well-being [5, 7].

Overall, research on stress in caregivers of children with NDDs has indicated that raising children with NDDs is especially stressful and that the current COVID-19 pandemic might increase stress further [3, 7]. Considering that exposure to stressful life events, family adversity, and ineffective parenting might contribute to chronic exacerbation of the symptoms of NDDs such as ADHD [17], which in turn might further increase the caregiver burden and negative effects on the family life, a continuing negative spiral is probable. The results of the present study might illustrate the NDD caregivers’ need for guidance and support during future emergencies (e.g., pandemics) to meet the needs of their children, mastering their new roles, and reduce interference with family life resulting from the emergency. A recent qualitative study, involving 15 respondents [38] found that new interpretations and an increased understanding of parents’ experiences are required to support parents who care for children with complex needs. Understanding parents’ experiences could reduce social isolation and exclusion and help to guide appropriate and supportive practices and services within and across medical, social, and family systems. Parents’ experience of managing complexities of care within health, social, and family systems remains particularly under-researched. Caregivers´ need for shared strategies to cope with and endure external stressors, such as the COVID-19 family life interference is important [3]. Based on our findings it is conceivable that the lack of support and guidance from schools, the Child, and Adolescents Mental Health Services (CAMHS), or other public agencies threatens the family life of caregivers of children with NDD by not providing them with the necessary tools and coping strategies [3, 7, 39]. Consistently with the Norwegian national caregiver survey [12] our findings seem to indicate that residing in more rural counties (i.e., Vestfold, and Telemark, and Rogaland) might decrease the risk for COVID-19 family life interference. Differences between caregivers in rural and urban settings may be explained by the fact that more rural counties are more likely to have local services offer particularly aimed to support the caregivers. However, the association was not consistent across the different counties. While a recently published scoping review [7] concluded that there is a lack of evidence-based studies and articles on this population, it also presented parenting advice for families with children with NDDs during the pandemic highlighting the importance of maintaining the child´s therapy and special educational routines, creating structured daily schedule with child-appropriate activities, and to keep in contact with children´ teachers and therapists [7]. However, further research is needed to explore the need for support, guidance, and coping strategies among caregivers of children with NDD and the associations of these needs with caregiver burden and family stress.

### Strengths and limitations

The cross-sectional design makes inferring causality not possible. However, testing for associations and differences between variables and groups, which is the purpose of this study, is possible with a cross-sectional design [40]. The use of a nonprobability convenience sampling such as strategic network sampling are prone to bias as respondents volunteering to participate through indirect contact such as posted notices on social media or web sites likely differs from those not volunteering. However, despite the risk of bias, the use of strategic network sampling provides unique access to the desired population. This is especially effective with the help of cooperating partners, such as patient representing organizations. Their unique access provides a platform for reaching people otherwise difficult to contact or identify [41]. The measures used in this study were not validated instruments which may have implications on the reliability [42]. However, this study is capturing what caregivers themselves emphasize as crucial perspectives in a high stress environment, which could be a foundation for future studies. Most of the variables included in the analysis were questions which were difficult to misinterpret. Taking into consideration the hereditary factor of NDDs [24] the use of a relatively short questionnaire with closed questions and a reduced number of options for answering was done to accommodate possible challenges related to executive functions and motivate to participate [18, 19]. In addition, open-ended questions were included to let the participants freely express themselves. The importance of the topic is demonstrated by the increasing research interest [7] and several different Norwegian user organizations on NDD, recommend the need investigating this area. This survey was developed by caregivers with lived experiences which we assume ensured the relevance of questions and helping us to understand the challenges and the beneficial aspects of the COVID-19 pandemic among the NDD caregiver population more clearly. The strengths and considerations presented above demonstrates the positive impact on outcomes when partnering with caregivers in all phases of the research process [43], while also providing us with a large sample size, another strong point of this study.

The output variable “COVID-19 Family Life Interference” was based on an open-ended question in which the respondents were able to express themselves freely, which we considered appropriate to render enough information to construct the output variable. Although this may indicate face validity [44], it is a limitation to our study that a validated instrument was not included to measure such an important concept. We cannot be certain that the output variable represents the COVID-19 family life interference, however the data was reasonably coherent across all the included variables. The use of measures developed by the user-team and not validated/psychometric tested is justified by the fact that no validated measure excited to capture conditions as experienced during the pandemic. The urgency to collect data during the pandemic while the respondents experienced it rather than recalling it, gave no time to develop such a validated instrument, and the potential for recall bias was reduced [45]. The development of such instruments should be conducted in future research. Despite these limitations it is our opinion that the overall results still provide an important contribution to the body of knowledge.

Research confidentiality [46] was very important for our user representatives; thus, the data did not include the diagnosis of the children. As a result, we were not able to separate subgroups according to different diagnoses, indicating that this sample may not be representative of the broader population of people with NDDs. However, collaborating organizations represent the children and adolescents diagnosed with Tourette’s syndrome, ADHD and ASD; it is therefore, reasonable to assume that people with these diagnoses are represented in this data. It was not specified in the written information about the survey whether one or both caregivers representing each household were allowed to participate and the importance of confidentiality which restricted the collection of demographic data hinders further examination of how many different households our population represents. Thus, the sample recruited for this study might be considered a limitation.

## Conclusion

“It is easy to tear down but takes a long time to rebuild” was a comment made by one of the team’s user representatives, with reference to the stable environment children with NDDs and their caregivers rely on. Both the children and the caregivers should be considered a group that is at risk and in need of continuous support and guidance as a family, especially during stressful life events such as the current COVID-19 pandemic.

Our study suggests that more information about stressful life event impacts should be provided to healthcare providers, social professionals, peers, people with NDDs, and caregivers of people with NDDs. In addition, there should be a focus on the identification of particularly vulnerable children and families, such as the population in this study, when preparing for future challenges. It is our hope that this study will inspire further exploration of this subject in collaboration with user representatives, so when the next pandemic or similar stressful life event occurs, groups at risk, such as people with NDD and their caregivers, are better equipped to face rather than fight the waves.

## Supplementary Information

**Additional file 1:** **Supplementary table**. Correlation matrix of independent variables - Checking for multicollinearity.

## Data Availability

The dataset generated during and analysed during the current study are not publicly available as information about public availability was not included in the participant information form but are available from the corresponding author on reasonable request.
