# Supplementary material for: Fighting the waves; Covid-19 family life interference in a neurodevelopmental disorder-caregiver population
Source: BMC Health Serv Res. 2022 Apr 10;22:472. doi: 10.1186/s12913-022-07836-3 (PMC8994698; doi:10.1186/s12913-022-07836-3)
Supplement: Supplementary file 1 — Additional file 1: Supplementary table. Correlation matrix of independent variables - Checking for multicollinearity. [file 12913_2022_7836_MOESM1_ESM.docx]

| ***Supplementary table*** *- Correlation matrix of independent variables - Checking for multicollinearity.*   \| Variable \| *1* \| *2* \| *3* \| 4 \| 5 \| 6 \| 7 \| 8 \| 9 \| 10 \| 11 \| 12 \| 13 \| 14 \| 15 \| 16 \| 17 \| 18 \| 19 \| 20 \| 21 \| 22 \| 23 \| 24 \| 25 \| 26 \| \| --- \| --- \| --- \| --- \| --- \| --- \| --- \| --- \| --- \| --- \| --- \| --- \| --- \| --- \| --- \| --- \| --- \| --- \| --- \| --- \| --- \| --- \| --- \| --- \| --- \| --- \| --- \| \| 1. Viken \| - \|  \|  \|  \|  \|  \|  \|  \|  \|  \|  \|  \|  \|  \|  \|  \|  \|  \|  \|  \|  \|  \|  \|  \|  \|  \| \| 2. Møre og Romsdal \| .537 \| - \|  \|  \|  \|  \|  \|  \|  \|  \|  \|  \|  \|  \|  \|  \|  \|  \|  \|  \|  \|  \|  \|  \|  \|  \| \| 3. Agder \| .483 \| .386 \| - \|  \|  \|  \|  \|  \|  \|  \|  \|  \|  \|  \|  \|  \|  \|  \|  \|  \|  \|  \|  \|  \|  \|  \| \| 4. Rogaland \| .569 \| .459 \| .412 \| - \|  \|  \|  \|  \|  \|  \|  \|  \|  \|  \|  \|  \|  \|  \|  \|  \|  \|  \|  \|  \|  \|  \| \| 5. Trøndelag \| .559 \| .440 \| .412 \| .475 \| - \|  \|  \|  \|  \|  \|  \|  \|  \|  \|  \|  \|  \|  \|  \|  \|  \|  \|  \|  \|  \|  \| \| 6. Vestland \| .607 \| .487 \| .433 \| .513 \| .500 \| - \|  \|  \|  \|  \|  \|  \|  \|  \|  \|  \|  \|  \|  \|  \|  \|  \|  \|  \|  \|  \| \| 7. Nordland \| .454 \| .359 \| .327 \| .382 \| .380 \| .406 \| - \|  \|  \|  \|  \|  \|  \|  \|  \|  \|  \|  \|  \|  \|  \|  \|  \|  \|  \|  \| \| 8. Troms og Finnmark \| .481 \| .388 \| .344 \| .407 \| .396 \| .437 \| .324 \| - \|  \|  \|  \|  \|  \|  \|  \|  \|  \|  \|  \|  \|  \|  \|  \|  \|  \|  \| \| 9.Vestfold og Telemark \| .533 \| .421 \| .386 \| .455 \| .452 \| .477 \| .361 \| .382 \| - \|  \|  \|  \|  \|  \|  \|  \|  \|  \|  \|  \|  \|  \|  \|  \|  \|  \| \| 10. Innlandet \| .541 \| .431 \| .391 \| .456 \| .451 \| .489 \| .372 \| .387 \| .431 \| - \|  \|  \|  \|  \|  \|  \|  \|  \|  \|  \|  \|  \|  \|  \|  \|  \| \| 11. Not responded \| .622 \| .501 \| .449 \| .526 \| .514 \| .563 \| .418 \| .449 \| .477 \| .503 \| - \|  \|  \|  \|  \|  \|  \|  \|  \|  \|  \|  \|  \|  \|  \|  \| \| 12. Home office \| .128 \| .084 \| .154 \| .104 \| .138 \| .086 \| .104 \| .084 \| .130 \| .103 \| .082 \| - \|  \|  \|  \|  \|  \|  \|  \|  \|  \|  \|  \|  \|  \|  \| \| 13. Lost income \| .002 \| -.011 \| .057 \| .049 \| .047 \| -.009 \| .030 \| .014 \| .017 \| .007 \| .042 \| .002 \| - \|  \| \|  \|  \|  \|  \|  \|  \|  \|  \|  \|  \|  \| \| 14. Concerned economy \| -.033 \| -.028 \| .004 \| -.039 \| .033 \| .004 \| -.007 \| .016 \| -.015 \| .019 \| .013 \| .105 \| -.381 \| - \|  \|  \|  \|  \|  \|  \|  \|  \|  \|  \|  \|  \| \| 15. Home schooling \| .006 \| .005 \| -.038 \| -.007 \| .001 \| .020 \| .004 \| .017 \| -.003 \| .023 \| .011 \| -.303 \| -.070 \| -.018 \| - \|  \|  \|  \|  \|  \|  \|  \|  \|  \|  \|  \| \| 16. Arr. Equipment \| .009 \| .031 \| -.004 \| .019 \| .020 \| -.019 \| .045 \| .022 \| .061 \| .020 \| -.005 \| -.008 \| -.034 \| -.014 \| -.022 \| - \|  \|  \|  \|  \|  \|  \|  \|  \|  \|  \| \| 17. Edu. support (1) \| .040 \| .008 \| -.011 \| .029 \| .041 \| .048 \| .034 \| -.002 \| .084 \| .094 \| .005 \| -.082 \| -.050 \| -.006 \| .037 \| .262 \| - \|  \|  \|  \|  \|  \|  \|  \|  \|  \| \| 18. Edu. Support (2) \| .032 \| .014 \| -.006 \| .016 \| -.008 \| .032 \| .010 \| .050 \| .064 \| .060 \| .033 \| -.037 \| -.019 \| .017 \| .066 \| .256 \| .519 \| - \|  \|  \|  \|  \|  \|  \|  \|  \| \| 19. Guidance home school \| .012 \| .059 \| .002 \| .069 \| .020 \| .035 \| -.043 \| .023 \| .040 \| -.030 \| .020 \| -.016 \| .018 \| .026 \| .010 \| -.061 \| .095 \| .289 \| - \|  \|  \|  \|  \|  \|  \|  \| \| 20. Support; school, \| .020 \| -.048 \| -.031 \| -.027 \| .007 \| -.006 \| .090 \| .003 \| .029 \| .022 \| -.029 \| -.014 \| -.001 \| -.053 \| .015 \| -.055 \| -.007 \| .097 \| -.295 \| - \|  \|  \|  \|  \|  \|  \| \| 21. Child anxious (1) \| -.007 \| .033 \| .099 \| .017 \| .003 \| .022 \| .033 \| .025 \| .036 \| .035 \| -.014 \| .048 \| .025 \| -.065 \| -.061 \| -.018 \| -028 \| .003 \| -.043 \| .012 \| - \|  \|  \|  \|  \|  \| \| 22. Child anxious (2) \| .006 \| .010 \| .000 \| .022 \| .010 \| .006 \| .040 \| .015 \| .051 \| .038 \| -.008 \| .066 \| .012 \| -.084 \| -.031 \| -.083 \| -.012 \| -.015 \| -.023 \| .031 \| .252 \| - \|  \|  \|  \|  \| \| 23. More children \| .001 \| -.007 \| .006 \| -.011 \| -.007 \| .007 \| -.002 \| .035 \| .025 \| -.041 \| -.021 \| .004 \| -.058 \| .031 \| -.157 \| .024 \| -.062 \| -.029 \| -.033 \| .010 \| .014 \| .011 \| - \|  \|  \|  \| \| 24. Increased conflicts (1) \| -.006 \| .025 \| .008 \| .010 \| -.005 \| -.009 \| .019 \| -.041 \| -.040 \| .044 \| .042 \| -.066 \| -.044 \| .098 \| -.050 \| -.070 \| .010 \| .027 \| .090 \| -.040 \| -.129 \| -.020 \| -.072 \| - \|  \|  \| \| 25. Increased conflicts (2) \| -.013 \| .029 \| -.028 \| -.028 \| -.068 \| .011 \| .018 \| -.006 \| -.139 \| .017 \| .072 \| -.019 \| -.024 \| .065 \| -.059 \| -.078 \| .042 \| .040 \| .001 \| .006 \| -.022 \| -.115 \| -.127 \| .411 \| - \|  \| \| 26. Respite care scheme \| -.006 \| -.059 \| .028 \| -.034 \| .040 \| -.043 \| -.015 \| -.072 \| -.031 \| -.052 \| -.014 \| .028 \| .033 \| .015 \| -.013 \| -.008 \| .005 \| -.031 \| .062 \| .082 \| -.050 \| -.014 \| .040 \| -.058 \| -.014 \| - \| |
| --- | --- | --- | --- | --- | --- | --- | --- | --- | --- | --- | --- | --- | --- | --- | --- | --- | --- | --- | --- | --- | --- | --- | --- | --- | --- | --- | --- | --- | --- | --- | --- | --- | --- | --- | --- | --- | --- | --- | --- | --- | --- | --- | --- | --- | --- | --- | --- | --- | --- | --- | --- | --- | --- | --- | --- | --- | --- | --- | --- | --- | --- | --- | --- | --- | --- | --- | --- | --- | --- | --- | --- | --- | --- | --- | --- | --- | --- | --- | --- | --- | --- | --- | --- | --- | --- | --- | --- | --- | --- | --- | --- | --- | --- | --- | --- | --- | --- | --- | --- | --- | --- | --- | --- | --- | --- | --- | --- | --- | --- | --- | --- | --- | --- | --- | --- | --- | --- | --- | --- | --- | --- | --- | --- | --- | --- | --- | --- | --- | --- | --- | --- | --- | --- | --- | --- | --- | --- | --- | --- | --- | --- | --- | --- | --- | --- | --- | --- | --- | --- | --- | --- | --- | --- | --- | --- | --- | --- | --- | --- | --- | --- | --- | --- | --- | --- | --- | --- | --- | --- | --- | --- | --- | --- | --- | --- | --- | --- | --- | --- | --- | --- | --- | --- | --- | --- | --- | --- | --- | --- | --- | --- | --- | --- | --- | --- | --- | --- | --- | --- | --- | --- | --- | --- | --- | --- | --- | --- | --- | --- | --- | --- | --- | --- | --- | --- | --- | --- | --- | --- | --- | --- | --- | --- | --- | --- | --- | --- | --- | --- | --- | --- | --- | --- | --- | --- | --- | --- | --- | --- | --- | --- | --- | --- | --- | --- | --- | --- | --- | --- | --- | --- | --- | --- | --- | --- | --- | --- | --- | --- | --- | --- | --- | --- | --- | --- | --- | --- | --- | --- | --- | --- | --- | --- | --- | --- | --- | --- | --- | --- | --- | --- | --- | --- | --- | --- | --- | --- | --- | --- | --- | --- | --- | --- | --- | --- | --- | --- | --- | --- | --- | --- | --- | --- | --- | --- | --- | --- | --- | --- | --- | --- | --- | --- | --- | --- | --- | --- | --- | --- | --- | --- | --- | --- | --- | --- | --- | --- | --- | --- | --- | --- | --- | --- | --- | --- | --- | --- | --- | --- | --- | --- | --- | --- | --- | --- | --- | --- | --- | --- | --- | --- | --- | --- | --- | --- | --- | --- | --- | --- | --- | --- | --- | --- | --- | --- | --- | --- | --- | --- | --- | --- | --- | --- | --- | --- | --- | --- | --- | --- | --- | --- | --- | --- | --- | --- | --- | --- | --- | --- | --- | --- | --- | --- | --- | --- | --- | --- | --- | --- | --- | --- | --- | --- | --- | --- | --- | --- | --- | --- | --- | --- | --- | --- | --- | --- | --- | --- | --- | --- | --- | --- | --- | --- | --- | --- | --- | --- | --- | --- | --- | --- | --- | --- | --- | --- | --- | --- | --- | --- | --- | --- | --- | --- | --- | --- | --- | --- | --- | --- | --- | --- | --- | --- | --- | --- | --- | --- | --- | --- | --- | --- | --- | --- | --- | --- | --- | --- | --- | --- | --- | --- | --- | --- | --- | --- | --- | --- | --- | --- | --- | --- | --- | --- | --- | --- | --- | --- | --- | --- | --- | --- | --- | --- | --- | --- | --- | --- | --- | --- | --- | --- | --- | --- | --- | --- | --- | --- | --- | --- | --- | --- | --- | --- | --- | --- | --- | --- | --- | --- | --- | --- | --- | --- | --- | --- | --- | --- | --- | --- | --- | --- | --- | --- | --- | --- | --- | --- | --- | --- | --- | --- | --- | --- | --- | --- | --- | --- | --- | --- | --- | --- | --- | --- | --- | --- | --- | --- | --- | --- | --- | --- | --- | --- | --- | --- | --- | --- | --- | --- | --- | --- | --- | --- | --- | --- | --- | --- | --- | --- | --- | --- | --- | --- | --- | --- | --- | --- | --- | --- | --- | --- | --- | --- | --- | --- | --- | --- | --- | --- | --- | --- | --- | --- | --- | --- | --- | --- | --- | --- | --- | --- | --- | --- | --- | --- | --- | --- | --- | --- | --- | --- | --- | --- | --- | --- | --- | --- | --- | --- | --- | --- | --- | --- | --- | --- | --- | --- | --- | --- | --- | --- | --- | --- | --- | --- | --- | --- | --- | --- | --- | --- | --- | --- | --- | --- | --- | --- | --- | --- | --- | --- | --- | --- | --- | --- | --- | --- | --- | --- | --- | --- | --- | --- | --- | --- | --- | --- | --- | --- | --- | --- | --- | --- | --- | --- | --- | --- | --- | --- | --- | --- | --- | --- | --- | --- | --- | --- | --- | --- | --- | --- | --- | --- | --- | --- | --- | --- | --- | --- | --- | --- | --- | --- | --- | --- | --- | --- | --- | --- | --- | --- | --- | --- | --- | --- | --- | --- | --- | --- |
